# Supplementary material for: A Likelihood Approach for Real-Time Calibration of Stochastic Compartmental Epidemic Models
Source: PLoS Comput Biol. 2017 Jan 17;13(1):e1005257. doi: 10.1371/journal.pcbi.1005257 (PMC5240920; doi:10.1371/journal.pcbi.1005257)
Supplement: S1 File — (TAR.GZ) [file pcbi.1005257.s014.tar.gz › HSPH_Online-SI-Revision/PF08/n10-mild/n10-mild_PF2_summary2.pdf]

peak -8	3w spec	3w cum	5w spec	5w cum	end	3w spec LOG	3w cum LOG	5w spec LOG	5w cum LOG	end LOG
mean	230.7	227.8	91.6	128.6	67.6	0.3	0.3	0.2	0.3	0.2
median	82.6	98.2	46.3	64.3	58.3	0.2	0.3	0.2	0.2	0.2
quantile .1	26.7	25.5	25.2	26.2	36.6	0.1	0.1	0.1	0.1	0.1
quantile .9	453.9	463.8	219.8	302.4	118.3	0.6	0.7	0.4	0.1	0.4
27	19	36	29	5						

peak -4	3w spec	3w cum	5w spec	5w cum	end	3w spec LOG	3w cum LOG	5w spec LOG	5w cum LOG	end LOG
mean	39.	37.1	38.7	35.8	71.8	0.2	0.2	0.2	0.2	0.2
median	31.7	27.6	33.	30.6	62.	0.2	0.1	0.2	0.1	0.2
quantile .1	13.5	14.5	19.5	14.6	38.9	0.1	0.1	0.1	0.1	0.1
quantile .9	52.5	46.7	61.2	50.4	114.3	0.3	0.3	0.3	0.1	0.4
26	30	29	27	0						

peak	3w spec	3w cum	5w spec	5w cum	end	3w spec LOG	3w cum LOG	5w spec LOG	5w cum LOG	end LOG
mean	63.7	44.	137.3	59.8	141.4	0.2	0.2	0.3	0.2	0.4
median	53.1	37.1	88.4	54.7	115.8	0.2	0.1	0.3	0.2	0.4
quantile .1	15.1	14.8	34.4	17.2	57.8	0.1	0.1	0.1	0.1	0.2
quantile .9	125.2	77.1	262.3	117.8	255.2	0.4	0.3	0.6	0.1	0.6
10	14	0	9	0						

peak +4	3w spec	3w cum	5w spec	5w cum	end	3w spec LOG	3w cum LOG	5w spec LOG	5w cum LOG	end LOG
mean	403.1	174.8	9722.5	234.9	439.3	0.5	0.4	0.7	0.4	0.6
median	151.2	105.3	269.8	136.6	258.7	0.4	0.3	0.6	0.4	0.6
quantile .1	63.3	44.	72.	52.2	78.4	0.2	0.2	0.2	0.2	0.3
quantile .9	564.8	261.4	1903.	420.3	668.6	0.8	0.6	1.3	0.2	0.9
0	0	0	0	0						

peak +8	3w spec	3w cum	5w spec	5w cum	end	3w spec LOG	3w cum LOG	5w spec LOG	5w cum LOG	end LOG
mean	11 214.7	20 246.2	16 563.6	28 730.3	44 576.1	0.8	0.8	1.1	0.8	1.
median	289.	258.5	512.9	272.6	451.6	0.6	0.6	0.8	0.7	0.8
quantile .1	78.	77.3	81.8	78.8	84.1	0.3	0.3	0.3	0.3	0.3
quantile .9	3572.3	2455.	13 219.	3538.6	5683.6	1.6	1.4	2.1	0.3	1.8
1	0	1	0	1	{}	{}	{}	{}	{}	{}
